# Hemodynamic Assessment in Bicuspid Aortic Valve Disease and Aortic Dilation: New Insights From Voxel-By-Voxel Analysis of Reverse Flow, Stasis, and Energetics

**DOI:** 10.3389/fbioe.2021.725113

**Published:** 2022-01-13

**Authors:** Patrick Geeraert, Fatemehsadat Jamalidinan, Fiona Burns, Kelly Jarvis, Michael S. Bristow, Carmen Lydell, Silvia S. Hidalgo Tobon, Benito de Celis Alonso, Paul W. M. Fedak, James A. White, Julio Garcia

**Affiliations:** ^1^ Department of Cardiac Sciences, Cumming School of Medicine, University of Calgary, Calgary, AB, Canada; ^2^ Stephenson Cardiac Imaging Centre, Libin Cardiovascular Institute, Calgary, AB, Canada; ^3^ Department of Radiology, University of Calgary, Calgary, AB, Canada; ^4^ Department of Radiology, Northwestern University, Chicago, IL, United States; ^5^ Department of Physics, Universidad Autonoma Metropolitana, Mexico City, Mexico; ^6^ Faculty of Mathematical and Physical Sciences, Benemerita Universidad Autonoma de Puebla, Puebla, Mexico; ^7^ Alberta Children’s Hospital Research Institute, University of Calgary, Calgary, AB, Canada

**Keywords:** 4D-flow imaging, bicuspid aortc valve, reverse flow, kinetic energy, flow stasis, parametric mapping

## Abstract

**Objectives:** Clinical management decisions surrounding ascending aorta (AAo) dilation in bicuspid aortic valve (BAV) disease benefit from personalized predictive tools. 4D-flow MRI may provide patient-specific markers reflective of BAV-associated aortopathy. This study aims to explore novel 4D-flow MRI parametric voxel-by-voxel forward flow, reverse flow, kinetic energy and stasis in BAV disease. We hypothesize that novel parametric voxel-by-voxel markers will be associated with aortic dilation and referral for surgery and can enhance our understanding of BAV hemodynamics beyond standard metrics.

**Methods:** A total of 96 subjects (73 BAV patients, 23 healthy controls) underwent MRI scan. Healthy controls had no known cardiovascular disease. Patients were clinically referred for AAo dilation assessment. Indexed diameters were obtained by dividing the aortic diameter by the patient’s body surface area. Patients were followed for the occurrence of aortic surgery. 4D-flow analysis was performed by a single observer in five regions: left ventricular outflow tract (LVOT), AAo, arch, proximal descending aorta (PDAo), and distal descending aorta (DDAo). In each region peak velocity, kinetic energy (KE), forward flow (FF), reverse flow (RF), and stasis were measured on a voxel-by-voxel basis. T-tests (or non-parametric equivalent) compared flow parameters between cohorts. Univariate and multivariate analyses explored associations between diameter and parametric voxel-by-voxel parameters.

**Results:** Compared to controls, BAV patients showed reduced stasis (*p* < 0.01) and increased RF and FF (*p* < 0.01) throughout the aorta, and KE remained similar. In the AAo, indexed diameter correlated with age (R = 0.326, *p* = 0.01), FF (R = −0.648, *p* < 0.001), RF (R = −0.441, *p* < 0.001), and stasis (R = −0.288, *p* < 0.05). In multivariate analysis, FF showed a significant inverse association with AAo indexed diameter, independent of age. During a median 179 ± 180 days of follow-up, 23 patients (32%) required aortic surgery. Compared to patients not requiring surgery, they showed increased KE and peak velocity in the proximal aorta (*p* < 0.01), accompanied by increased RF and reduced stasis throughout the entire aorta (*p* < 0.01).

**Conclusion:** Novel voxel-by-voxel reverse flow and stasis were altered in BAV patients and are associated with aortic dilation and surgical treatment.

## 1 Introduction

Bicuspid aortic valve (BAV) is considered the most common congenital valvular malformation with an overall prevalence in the general population of 0.5–2% (Siu and Silversides, 2010). BAV disease includes heterogeneous morphological phenotypes of fused cusps and raphe (Sievers and Schmidtke, 2007) which can lead to different pathological and clinical outcomes (Masri et al., 2017). At a population level, BAV is associated with increased risk of aortic dilation (Jamalidinan et al., 2020), requiring surgical intervention in 30–50% of individuals (Branchetti et al., 2014). Despite this, there is still variability in the clinical management guidelines (Baumgartner et al., 2017; Borger et al., 2018) for BAV aortopathy and strategies among cardiovascular surgeons vary substantially (Verma et al., 2013). This may be related to current recommendations relying on binary thresholds of ascending aorta (AAo) diameter to guide timing of proximal aortic aneurysm surgery, though these are recognized as poor predictors of acute aortic events (Pape et al., 2007). Thus, much emphasis has been placed on identifying alternative, patient-specific markers of BAV aortopathy that may provide improved characterization for this patient population.

Abnormal hemodynamics within the aorta related to abnormal valve geometry is considered an important factor in the development of BAV aortopathy (Verma and Siu, 2014). Four-dimensional flow magnetic resonance imaging (4D-flow MRI) has become recognized technique to quantify and characterize abnormal hemodynamics in BAV in a recently published consensus (Michelena et al., 2021). Particularly, wall shear stress (WSS) and flow displacement have shown encouraging associations with aortic dilation (Mahadevia et al., 2013; Guzzardi et al., 2015; Garcia et al., 2016; van Ooij et al., 2017; Rodríguez-Palomares et al., 2018; Dux-Santoy et al., 2019). In addition, other flow-based parameters such as forward flow (FF), reverse flow (RF), and energetics can be easily derived from the 4D-flow MRI velocity field (Harloff et al., 2010; Jamalidinan et al., 2020). However, most of these flow-based parameters are derived using 2D plane-based approaches (Stalder et al., 2008), which have been shown to underestimate true values (Shen et al., 2018), and it does not take advantage of the 3D nature of 4D-flow MRI. Shen et al.(Shen et al., 2018) and Jarvis et al. (Jarvis et al., 2020; Jarvis et al., 2021) recently introduced 3D voxel-by-voxel methods that provide 4D-flow derived parametric mapping of FF, RF, kinetic energy (KE) and stasis in the thoracic aorta. In this study, we applied 4D-flow derived parametric mapping to: (Siu and Silversides, 2010): evaluate differences in 3D-derived aortic parametric voxel-by-voxel hemodynamic markers between BAV patients and healthy controls, (Sievers and Schmidtke, 2007), explore associations between these markers and a structural marker of aortopathy (i.e. aortic diameter), and (Masri et al., 2017) observe differences in these markers for patients progressing to aortic surgery versus patients not requiring surgery during observational follow-up. We hypothesize that novel parametric voxel-by-voxel markers will be associated with aortic dilation and referral for surgery and can enhance our understanding of BAV hemodynamics beyond standard metrics.

## 2 Materials and Methods

### 2.1 Study Cohort

Aortic 4D-flow MRI was acquired in 73 BAV patients (age = 49 ± 16 years; 21 female) and 23 healthy controls (age = 37 ± 14 years; eight female). Patients were recruited as a pre-defined sub-study of a prospective observational clinical outcomes registry at our institution. The study was coordinated by commercial software (cardioDI^TM^, Cohesic Inc, Calgary, Alberta) for the routine capture of patient informed consent, health questionnaires and for standardized collection of MRI-related variables. Healthy volunteers ≥18 years of age were recruited and underwent identical workflow and were required to have no known cardiovascular disease, hypertension or diabetes and have no contraindications for MRI (Kramer et al., 2013).

All subjects enrolled to our study were required to be ≥18 years of age and agree to the incremental performance of research pulse sequences inclusive of 4D-flow MRI, and prospective follow-up using electronic health data matching for iterative capture of clinical and procedural events. For this study patients were identified by standardized coding of clinical referral indications for BAV plus confirmation of BAV morphology by MRI. Patients were excluded for the following reasons: history of prior myocardial infarction or known non-ischemic cardiomyopathy, complex congenital heart disease, MRI-coded moderate-severe mitral insufficiency, or a left ventricle ejection fraction (LVEF) < 50%.

The study was approved by the Institutional Review Board (IRB) at our institution and all subjects provided written informed consent. All research activities were performed in accordance with the Declaration of Helsinki.

### 2.2 Cardiac Magnetic Resonance Data Acquisition

All MRI examinations were performed using 3T MRI scanners [Prisma (N = 71) or Skyra (N = 25), Siemens, Erlangen, Germany]. Indication-based protocolling ensured consistent imaging procedures for all subjects. Imaging was performed in accordance with published recommendations (Kramer et al., 2013). Routine, retrospectively-gated balanced steady-state free precession (SSFP) cine imaging was performed in 4-chamber, 3-chamber, and 2-chamber, sequential short axis ventricular views and short axis aortic valve views, the latter to characterize valve morphology. Through-plane 2D phase-contrast (2DPC) flow imaging of the aortic valve was performed at the valve annulus, cusp tips, and 1 cm below the annulus. 3D magnetic resonance angiography (MRA) of the thoracic aorta was performed using administration of 0.2 mmol/kg gadolinium contrast (Gadovist, Bayer, Canada). Approximately 5–10 min following contrast administration, retrospectively ECG-gated 4D-flow MRI (WIP 785A) was acquired during free breathing using navigator gating of diaphragmatic motion (Garcia et al., 2021). 4D-flow imaging parameters were as follows: spatial resolution (row × column × slice) = 2.0–2.5 × 2.0–2.5 × 2.4–3.5 mm^3^, temporal resolution = 36.24–40.56 m s, flip angle = 15; field of view (FOV) = 240–350 × 320–400 mm^2^, bandwidth = 455–495 Hz/Pixel, velocity sensitivity (Venc) = 150–550 cm/s, echo time = 2.01–2.35 m s, pulse repetition time = 4.53–5.07 m s.

### 2.3 4D-Flow Analysis

**FIGURE 1:**
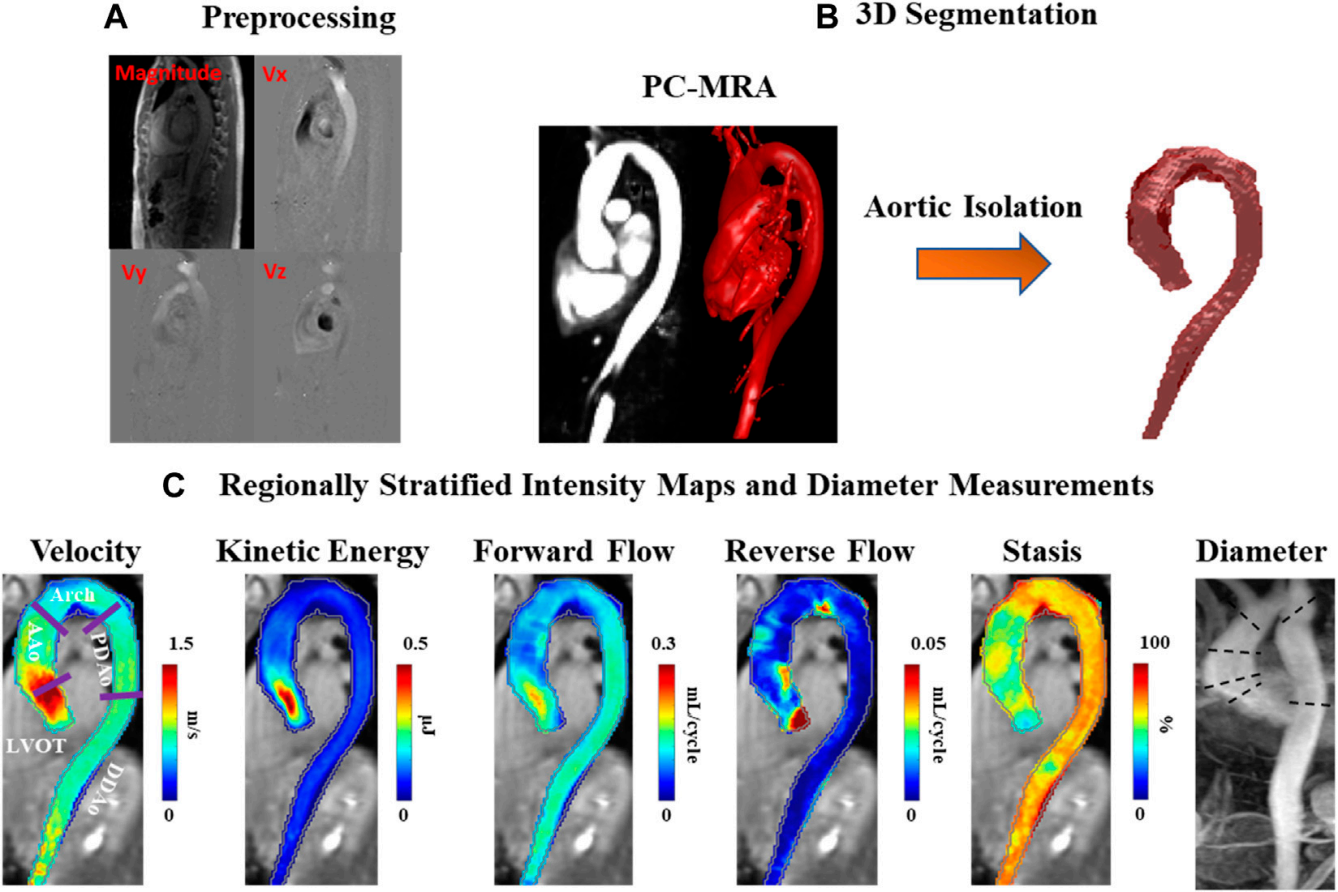
Workflow diagram. **(A)** Preprocessing of the original 4D-flow data, including calculation of 3D PC-MRA from the measured 3D velocities and magnitude. **(B)** 3D segmentation of the thoracic aorta. **(C)** Velocity, kinetic energy, forward flow, reverse flow, and stasis maps stratified by regions: left ventricular outflow tract (LVOT), ascending aorta (AAo), aortic arch (Arch), proximal descending aorta (PDAo) and distal descending aorta (DDAo). Maximum diameter calculated in each region using 2D PC-MRA.

4D-flow MRI analysis was performed using an in-house program developed in MATLAB (2020b) with workflow schematically summarized in Figure 1. Workflow consists of a preprocessing step through which raw data are subject to noise masking, velocity anti-aliasing, and corrections for Maxwell terms and eddy currents (Markl et al., 2007) (Figures 1–A). A time-averaged 3D phase-contrast MR angiography (PC-MRA) was derived from 4D-flow data, as previously described in the literature (Bock et al., 2007; Markl et al., 2007) to depict the aortic lumen region. Segmentation of the entire aorta was achieved using a semiautomatic software package called “4D-flow Analysis Tool” (Fatehi Hassanabad et al., 2020), which was previously developed in our group and is shown in Figures 1–B.

### 2.4 Data Analysis (Parametric Hemodynamic Maps)

The segmentation results were used to mask the 4D-flow velocity field and perform volumetric hemodynamic analyses. Parametric hemodynamic mapping of FF, RF, flow stasis, and KE was performed using an in-house MATLAB program according to a recently reported workflow (Shen et al., 2018; Jarvis et al., 2020; Jarvis et al., 2021). Briefly, 4D-flow data were interpolated to isotropic voxels (1 mm × 1 mm × 1 mm) using cubic spline interpolation. Each voxel was matched to the nearest plane (based on the shortest 3D distance) along the aortic centerline. Plane position along the centerline was used to define the direction of FF (i.e. flow moving downstream with respect to the plane) and RF (i.e. flow moving upstream with respect to the plane) for each voxel in the aortic volume (Shen et al., 2018). FF and RF were calculated for each aortic voxel, and time-frame, and summed over the cardiac cycle (leading to units of mL/cycle).

Voxel-wise flow stasis was determined as the percent of cardiac time-frames below the threshold value of velocity = 0.1 m/s (which was considered slow flow) (Jarvis et al., 2020). Voxel-wise KE was determined for each time-frame by:

$$KE\;=\;\frac{1}{2}\;\cdot \rho \cdot \;dV\;\cdot v{\left(t\right)}^{2}$$ (1)

with ρ being the blood density (assumed as 1,060 kg/m^3^) and *dV* the unit voxel volume (i.e. 1 mm^3^), summed over the cardiac cycle. Peak velocity was the maximum absolute velocity over systole using maximum intensity projections (MIPs) (Rose et al., 2016). Parametric maps were calculated as mean intensity projections for FF, RF, stasis, and KE, Figures 1–C.

Regional analysis was performed by dividing the aorta into five volumetric regions based on standard anatomic landmarks recommended in the thoracic aortic disease guidelines and previous studies (Fatehi Hassanabad et al., 2020; Hiratzka et al., 2010; Garcia et al., 2017; Garcia et al., 2015): 1) left ventricular outflow tract (LVOT); 2) ascending aorta (AAo); 3) aortic arch (Arch); 4) proximal descending aorta (PDAo); 5) distal descending aorta (DDAo) (Figures 1–C). All aforementioned flow parameters were calculated separately in each of these regions. Reynolds number was calculated at the aortic vena contracta location to assess flow regime (Stalder et al., 2011).

### 2.5 Valve Phenotypes, Aortic Diameters, and Dilation Patterns

**FIGURE 2:**
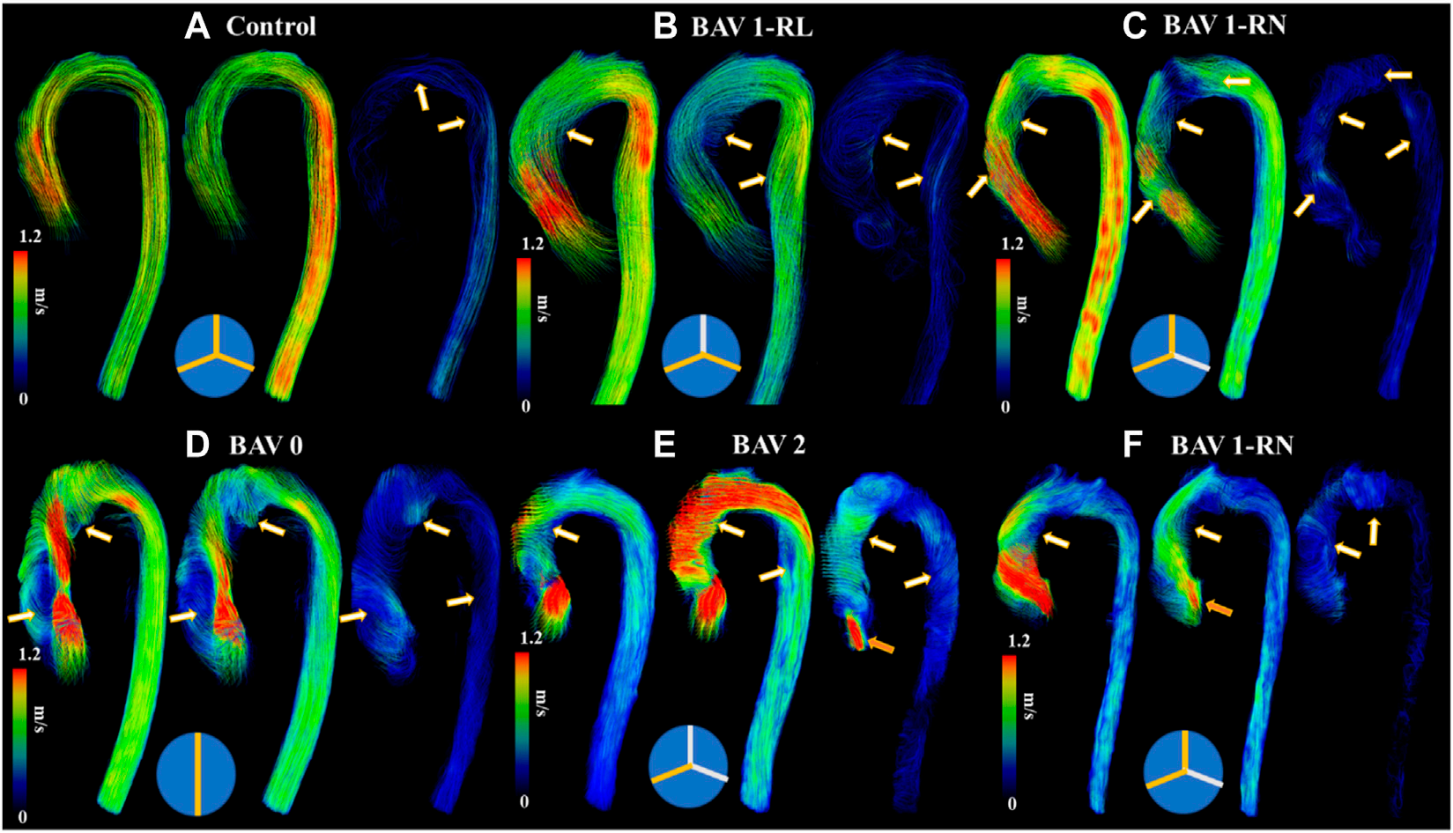
Helical flow patterns throughout the cardiac cycle. Panel **(A)** shows a control. Panel **(B)** shows a bicuspid aortic valve (BAV) with right-left (RL) fusion. Panel **(C)** shows a BAV with right-non coronary (RN) fusion. Panel **(D)** shows a BAV type 0 fusion. Panel **(E)** shows a BAV type 2 fusion with moderate aortic regurgitation. Panel **(F)** shows a BAV RN fusion with mild aortic regurgitation. White-orange arrows point to helical flow patterns and orange arrows to aortic valve regurgitation jet. Note that BAV patients develop reverse helical flow in the ascending aorta. At the bottom of each case a diagram of Sievers fusion type is illustrated.

BAV phenotypes were evaluated from aortic valve cine SSFP image acquisitions and categorized according to Sievers’ classifications (Sievers and Schmidtke, 2007), as follows: type 0 (no raphe); type 1 RL (one raphe connecting the right coronary and left coronary cusps); type 1 RN (one raphe connecting the right coronary and non-coronary cusps); type 2 (two raphes), Figure 2.

Maximum aortic diameters (mm) were measured for each aortic region using 3D contrast-enhanced MRA data, as recommended by published guidelines (Hiratzka et al., 2010). Diameters were indexed to body surface area (BSA) (Gehan and George, 1970) to normalize data across both study and volunteer populations. BSA-indexed diameters have been suggested to offer enhanced prognostic value for BAV patients compared to raw diameter values (Davies et al., 2006).

In keeping with previously used classifications (González-Santos and Arnáiz-García, 2017), patients were stratified by AAo dilation severity, as follows: non-dilated (max AAo diameter <35 mm), moderately dilated (35 mm < max AAo diameter <45 mm), and severely dilated (max AAo diameter >45 mm).

### 2.6 Statistical Analysis

A Shapiro Wilks test was used to determine the normality of each parameter. A student’s t-test (or non-parametric equivalent if at least one parameter had a non-normal distribution) was used to evaluate differences in parameter means between two opposing cohorts; when analyzing differences between more than two opposing cohorts, a one-way ANOVA (followed by Tukey’s post hoc test) was used.

To determine associations between aortic hemodynamic measures and vessel dilation, we correlated aortic diameters to each hemodynamic parameter within the BAV cohort only. Pearson correlations (or Spearman if at least one parameter had a non-normal distribution) were used and correlations were performed in the AAo region alone.

To further elucidate what hemodynamic parameters are most significantly associated with aortic remodelling in the context of BAV disease, a multivariate model was constructed including BSA-indexed aortic diameter as the dependent variable with age and hemodynamic parameters as independent variables. In each model, hemodynamic parameters were only included if they demonstrated a significant univariate association with the indexed aortic diameter and no multicollinearity with each other; however, age was always included to evaluate its importance in aortic remodelling relative to flow parameters.

For all tests, a *p*-value ≤ 0.05 was considered statistically significant. For univariate analyses, a correlation coefficient greater than absolute 0.25 was additionally required to be considered statistically significant. All statistical analyses were performed in SPSS 25 (Chicago, IL).

## 3 Results

### 3.1 Cohort Assignments

Of the BAV patients, there were: Type 0 (*n* = 19), Type 1 (*n* = 47; 11 RN, 36 RL), Type 2 (*n* = 4), and un-identified (*n* = 3). Patients with type 2 or un-identified valve phenotype were not included in statistical analyses due to the small sample size. Normal aortic valve morphology was confirmed in all healthy volunteers.

Twelve patients (16.4%) had no aortic dilation, 24 (32.8%) met moderate dilation criteria, and 27 (37.9%) met severe dilation criteria. Of those with dilation, a root morphotype was observed in 10 (19.6%) patients, while an ascending morphotype was observed in 13 (25.5%). No aortic dilation criteria was met in any of the health volunteers.

### 3.2 Subject Demographics and Aortic Dimensions

Baseline patient characteristics are provided in Table 1; standard aortic measurements are provided separately in Table 2. Compared to healthy volunteers (i.e. percent change in mean value), BAV patients were significantly older ([24%]; *p* < 0.01). However, no significant differences in left ventricle (LV) chamber volumes, mass or function were identified. Aortic measurements showed larger diameters of the AAo ([31%], *p* < 0.01), PDAo ([15%], *p* < 0.05), and DDAo ([11%], *p* < 0.01]) among patients with BAV disease. Once diameter was indexed to BSA, these differences only persisted at the AAo ([24%], *p* < 0.01) location.

**TABLE 1:** Demographics of study cohort.

Parameter	Cohorts									
	All subjects		BAV types			Dilation severity			Outcome	
	Control (*n* = 23)	BAV (*n* = 73)	Type 1-RL (*n* = 36)	Type 1-RN (*n* = 11)	Type 0 (*n* = 19)	Non dilation (*n* = 12)	Mod. Dilation (*n* = 24)	Severe dilation (*n* = 27)	No Surgery (*n* = 49)	Surgery (*n* = 24)
Age (yrs)	37 ± 14	49 ± 16*	49 ± 17	53 ± 14	45 ± 15	38 ± 19	50 ± 13	50 ± 15	48 ± 17	51 ± 13
Female (n)	8 (35%)	21 (29%)	9 (25%)	4 (36%)	5 (26%)	8 (67%)	3 (13%)†	5 (19%)‡	17 (35%)	4 (17%)
BSA (m2)	1.9 ± 0.3	2.0 ± 0.3	2.0 ± 0.2	2.0 ± 0.3	2.0 ± 0.3	1.8 ± 0.3	2.1 ± 0.2†	2.0 ± 0.2‡	2.0 ± 0.3	2.1 ± 0.2
Heart Rate (bpm)	64 ± 11	63 ± 12	65 ± 12	57 ± 5	62 ± 12	62 ± 10	66 ± 14	62 ± 11	63 ± 13	63 ± 9
LVEDV (ml)	167 ± 40	189 ± 63	190 ± 67	177 ± 37	169 ± 56	159 ± 29	198 ± 55	206 ± 73	175 ± 48	210 ± 78*
LVESV (ml)	64 ± 19	76 ± 34	78 ± 33	59 ± 22	68 ± 32	63 ± 16	81 ± 32	85 ± 41	72 ± 29	80 ± 40
LVEF (%)	62 ± 5	60 ± 9	59 ± 10	67 ± 9	61 ± 7	61 ± 4	59 ± 13	60 ± 9	59 ± 10	63 ± 7
LV Mass (g)	103 ± 31	132 ± 49	127 ± 48	164 ± 63	117 ± 32	105 ± 26	142 ± 42	144 ± 61	120 ± 38	153 ± 60*
LVCO (L/min)	6.7 ± 1.7	7.2 ± 2.7	7.3 ± 2.9	6.6 ± 1.4	6.5 ± 1.7	6.0 ± 0.8	7.8 ± 3.0	7.7 ± 3.0	6.5 ± 2.2	8.5 ± 3.0*

BAV- bicuspid aortic valve, Mod.- moderate, Morpho.—morphotype, BSA-body surface area, LV—center ventricular, LVEDV- center ventricular end-diastolic volume, LVESV—center ventricular end-systolic volume, LVEF—center ventricular ejection fraction, LVCO- center ventricular cardiac output. *p < 0.05 between opposing cohorts, †*p* < 0.05 between mod. dilation and non-dilation cohorts, and ‡*p* < 0.05 between severe dilation and non-dilation cohorts.

**TABLE 2:** Parameter differences between BAV patients and healthy controls at each aortic region.

Parameter	Location									
	LVOT		AAo		Arch		PDAo		DDAo	
	Control	BAV	Control	BAV	Control	BAV	Control	BAV	Control	BAV
Diameter (mm)	28 ± 5	29 ± 4	28 ± 4	40 ± 7**	25 ± 3	26 ± 5	20 ± 3	23 ± 4*	18 ± 3	20 ± 3*
Indexed Diameter (mm/m^2^)	15 ± 2	14 ± 2	15 ± 3	20 ± 4**	13 ± 2	13 ± 3	11 ± 2	11 ± 2	10 ± 2	10 ± 2
Peak Velocity (m/s)	1.3 ± 0.2	1.9 ± 1.0*	1.5 ± 0.3	2.6 ± 1.2**	1.2 ± 0.2	1.5 ± 0.8	1.2 ± 0.3	1.3 ± 0.8	1.4 ± 0.4	1.2 ± 0.5
KE (μJ)	1.9 ± 0.5	2.6 ± 1.8	1.8 ± 0.6	2.3 ± 1.2	1.5 ± 0.6	1.7 ± 1.0	1.8 ± 0.7	1.7 ± 1.1	2.0 ± 0.9	1.6 ± 0.9
FF (mL/cycle)	0.18 ± 0.04	0.15 ± 0.06*	0.18 ± 0.04	0.12 ± 0.03**	0.17 ± 0.04	0.14 ± 0.04*	0.19 ± 0.05	0.16 ± 0.05	0.22 ± 0.07	0.17 ± 0.05*
RF (mL/cycle)	0.025 ± 0.01	0.045 ± 0.05*	0.011 ± 0.001	0.039 ± 0.02**	0.010 ± 0.006	0.029 ± 0.02**	0.010 ± 0.01	0.019 ± 0.02**	0.005 ± 0.004	0.015 ± 0.025*
Stasis (%)	33 ± 9	22 ± 12**	50 ± 10	23 ± 11**	52 ± 10	32 ± 16**	53 ± 10	35 ± 18**	43 ± 13	38 ± 17

Diameter and indexed diameter measurements at the SOV, region are not included. LVOT-center ventricular outflow tract, AAo-ascending aorta, Arch—aorta arch, PDAo-proximal descending aorta, DDAo—distal descending aorta, BAV- bicuspid aortic valve, KE—kinetic energy, FF—forward flow, RF—reverse flow. Values are reported as mean ± stdev. *p < 0.05 and **p < 0.001.

Compared to Type 1 BAV subjects, Type 0 BAV subjects were associated with lower indexed diameters of the SOV ([-4%], *p* < 0.05). No other significant differences between valve types were identified. BAV patients with moderate or severe dilation had significantly larger BSAs and a smaller proportion of females when compared with non-dilated BAV patients. There were no other significant differences in baseline characteristics between dilation severity classifications.

### 3.3.4D-Flow MRI Findings

#### 3.3.1 BAV Patients vs Healthy Controls

**FIGURE 3:**
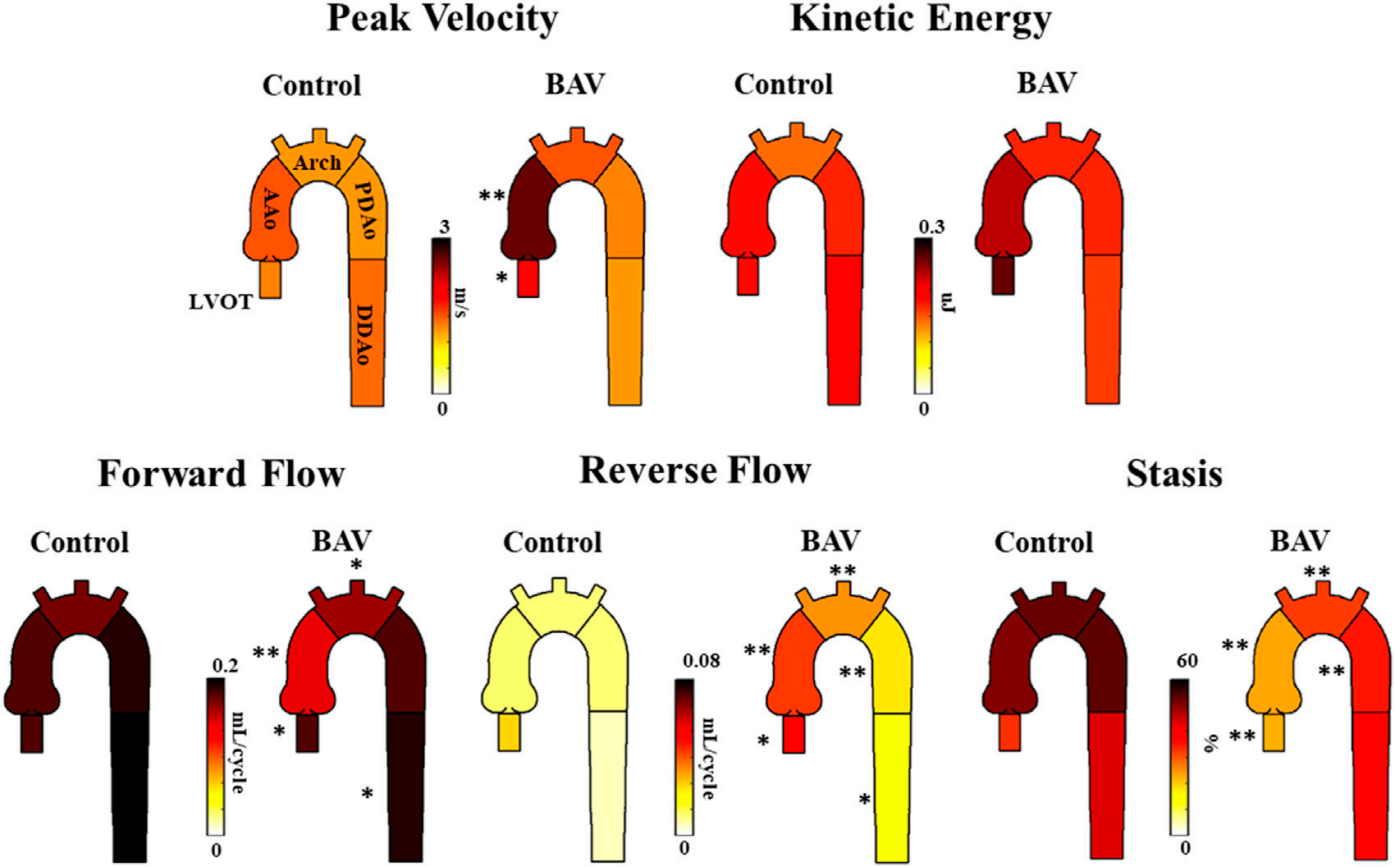
Differences in hemodynamic parameters between BAV patient cohort (right) and healthy control cohort (left). Note: BAV—bicuspid aortic valve, LVOT—left ventricular outflow tract, AAo—ascending aorta, Arch**-**aortic arch, PDAo—proximal descending aorta, DDAo**-**distal descending aorta. Symbols indicate significant *p*-values: * *p* < 0.05 ** *p* < 0.001.

It was possible to observe small helical patterns in the arch and in the proximal descending aorta, Figure 2. BAV patients exhibited larger helical flow regions with pronounced reverse flow direction in the ascending aorta, Figure 2-Bottom. Peak velocity measurements were greater in BAV patients at the LVOT ([32%], *p* < 0.05) and AAo ([42%], *p* < 0.001) while FF was lower in the AAo ([-33%, *p* < 0.05) (Table 2; Figure 3). The BAV cohort also showed significantly elevated RF throughout the entire aorta, with the largest increase being at the AAo ([254%], *p* < 0.001). Stasis levels were significantly lower in BAV patients from the LVOT to PDAo, with the largest decrease being at the AAo ([-54%], *p* < 0.001). Controls had Reynolds numbers in the range of [5,275–1,135] while BAV patients had a range of [5,504–3,0411] at vena contracta; thus indicating the presence of turbulent flow in the ascending aorta.

#### 3.3.2 Bicuspid Aortic Valve Types

**FIGURE 4:**
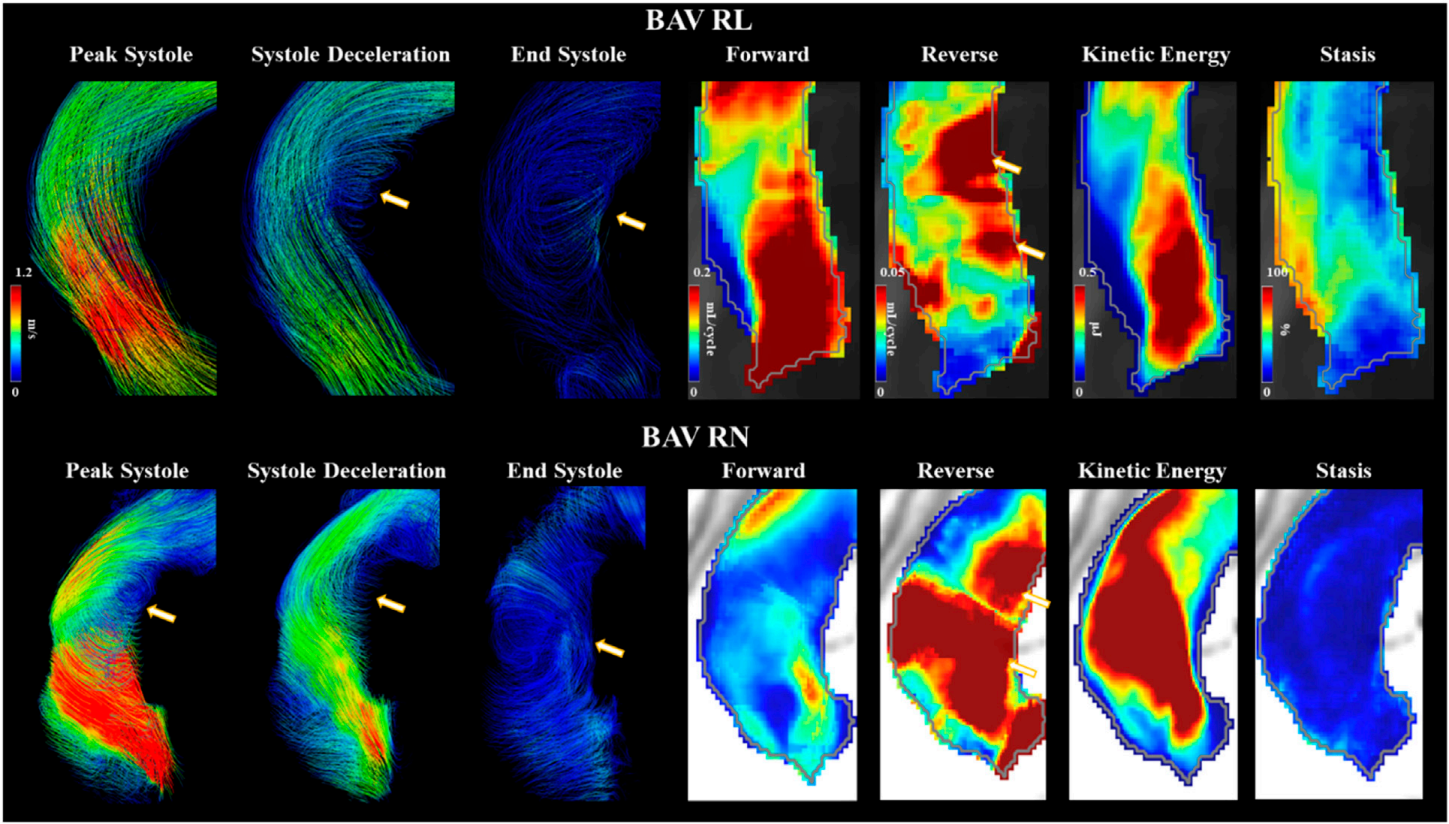
Bicuspid aortic valve cases with right-left and right-non coronary fusion. Arrows point to helical flow patterns. Forward flow, reverse flow, kinetic energy, and flow stasis from each subject are represented using maximum intensity projections. Gray line represents the vessel wall from the aortic segmentation.

Type 0 BAVs exhibited less stasis in the DDAo (30 ± 18% vs 41 ± 16% [-27%]; *p* < 0.05) compared to all Type 1 BAVs together. No other significant difference was found in the comparison of Type 0 and Type 1 valves. When accounting for specific phenotypes of Type 1 BAVs, Type 1-RN possessed the greatest peak velocity in the arch when compared to both Type 1-RL (2.1 ± 1.4 m/s vs 1.4 ± 0.5 m/s [50%]; *p* < 0.05) and Type 0 (2.1 ± 1.4 m/s vs 1.4 ± 0.4 m/s [50%]; *p* < 0.05). No other results were statistically significant. An example of Type 1-RL hemodynamics is illustrated in Figure 4.

#### 3.3.3 Dilation Severity

Moderately dilated patients showed less stasis in the AAo ([-36%], *p* < 0.05) and more RF in the arch ([83%], *p* < 0.05) compared to non-dilated (Table 3). Severely dilated patients showed less stasis in the AAo ([-39%], *p* < 0.01) and arch ([-34%], *p* < 0.05) accompanied by more RF in the arch ([106%], *p* < 0.01) compared to non-dilated patients. No other significant differences in these cohorts were observed.

**TABLE 3:** Parameter differences between BAV dilation severity.

Parameter	AAo			Arch		
	Non-dilated (*n* = 22)	Mod. dilation (*n* = 24)	Severe Dilation (*n* = 27)	Non-dilated (*n* = 22)	Mod. dilation (*n* = 24)	Severe Dilation (*n* = 27)
RF (mL/cycle)	0.033 ± 0.015	0.037 ± 0.016	0.045 ± 0.016*	0.018 ± 0.010	0.027 ± 0.017*	0.039 ± 0.018**
Stasis (%)	31 ± 13	20 ± 9*	19 ± 10**	41 ± 15	33 ± 17	27 ± 14*

Mod.—moderate; RF- reverse flow. Values are reported as mean ± stdev. *: *p* < 0.05 compared with non-dilated, **p < 0.01 compared with non-dilated.

### 3.4 Univariate and Multivariate Associations

**FIGURE 5:**
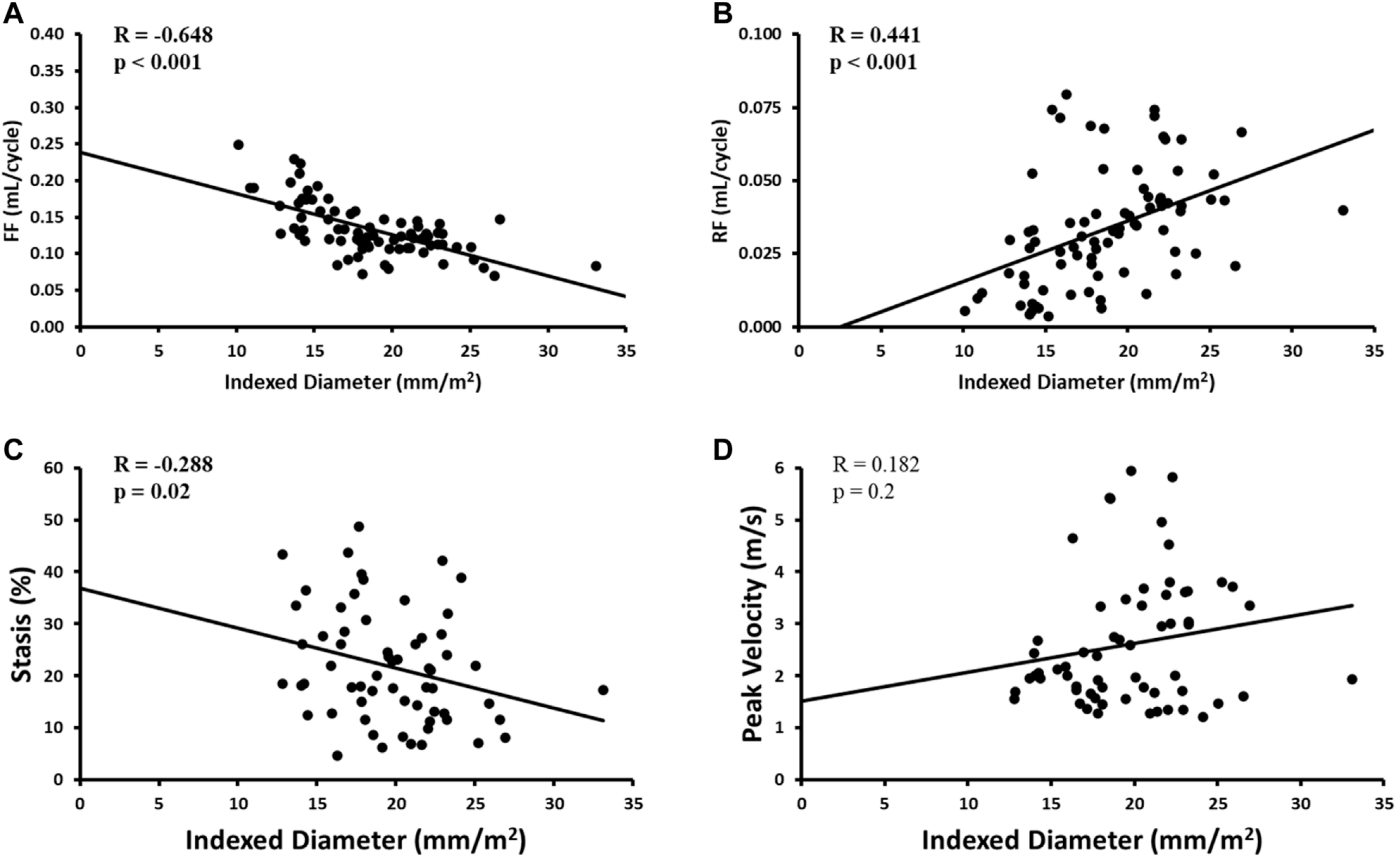
Correlates of aortic diameter in the AAo region only. **Panel A** shows forward flow (FF) plot. **Panel B** shows reverse flow (RF) plot. **Panel C** shows stasis plot. **Panel D** shows peak velocity plot.

Indexed AAo diameter correlated positively with age (R = 0.326, *p* = 0.01), but negatively with FF (R = -0.648, *p* < 0.001), RF (R = 0.441, *p* < 0.001) and stasis (R = −0.288, *p* < 0.05). No other significant univariate correlations were found between indexed diameter and hemodynamics at the level of the AAo. Flow-related associations are shown in Figure 5.

Multivariate results are shown in Table 4. Focusing on the AAo, the model included indexed AAo diameter as the dependent variable and age, AAo FF, AAo stasis, and AAo peak velocity as the independent variables. This model demonstrated an overall R = 0.709 and showed AAo FF (*β* = -0.492, *p* = 0.001) to be most strongly associated with indexed AAo diameter.

**TABLE 4:** Multiple linear regression results.

Model	Associations	
	Std. Coef	
AAo diameter indexed	β	*p*
Age	0.174	0.080
AAo FF	−0.492	0.001
AAo RF	0.198	0.151
AAo stasis	−0.008	0.954
AAo Peak Velocity	0.031	0.778

Model only included significant univariate correlations (R > 0.25, *p* < 0.01) and no multicollinearity with each other. AAo—ascending aorta, FF- forward flow, RF- reverse flow, *ß* - standardized coefficient, S.E., standard error.

### 3.5 Surgical Intervention

Twenty-four patients underwent surgical intervention and had a follow-up of 179 ± 180 days. Of these patients, 18 (78%) had an aortic valve replacement, two (9%) had an AAo replacement, and three (13%) had both. Patients who received surgery had larger diameters at the arch ([14%], *p* < 0.01), greater LV mass ([27%], *p* < 0.05), and greater LVCO ([31%], *p* < 0.01) than those who did not receive surgery. No other significant differences in baseline characteristics were found.

**FIGURE 6:**
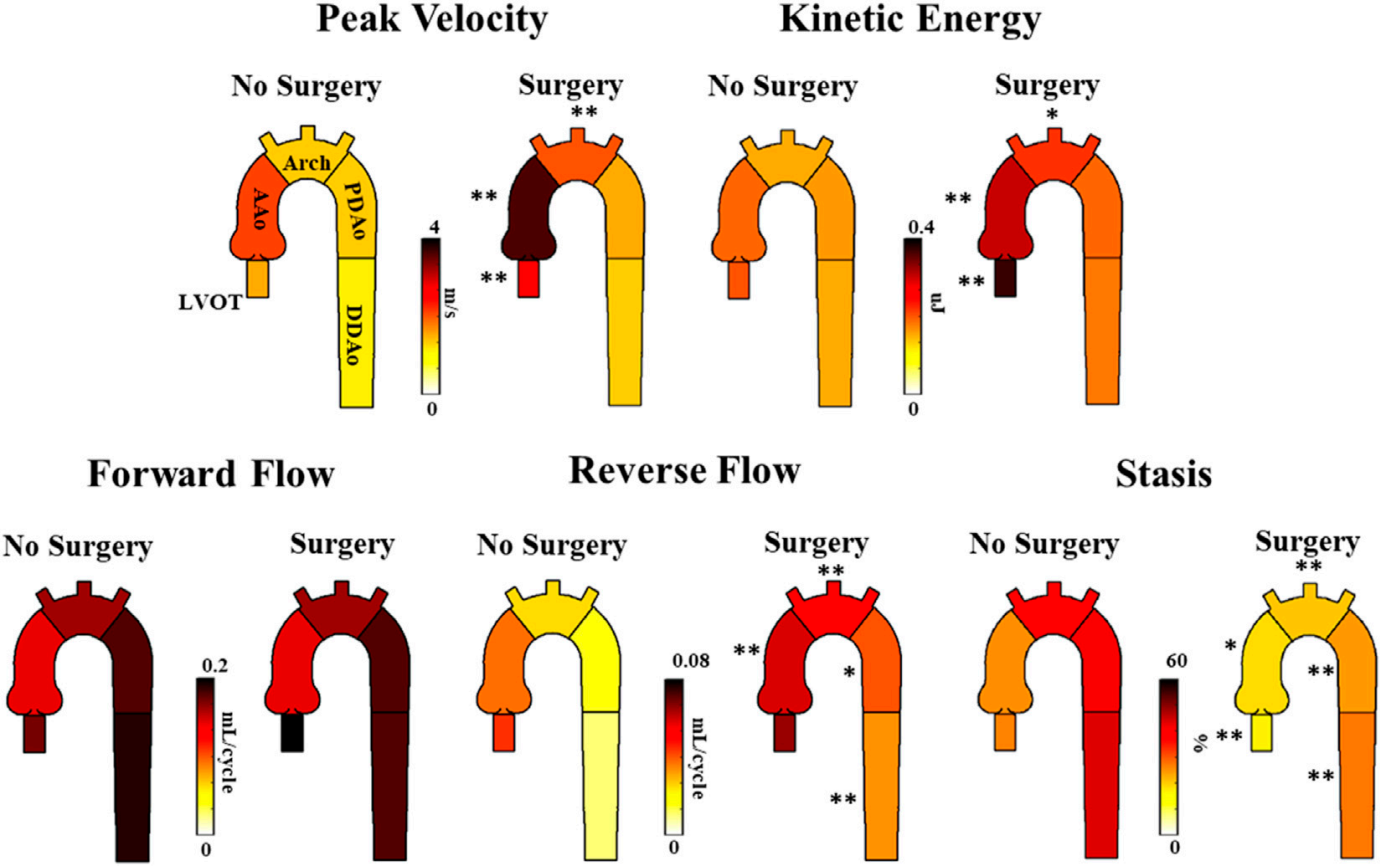
Differences in hemodynamic parameters between BAV patients who received follow-up surgery and BAV patients who did not. Note: BAV—bicuspid aortic valve, LVOT—left ventricular outflow tract, AAo—ascending aorta, Arch—aortic arch, PDAo—proximal descending aorta, DDAo—distal descending aorta. Symbols indicate significant *p*-values: * *p* < 0.05 ** *p* < 0.001.

Patients with surgical intervention demonstrated greater KE and peak velocity at the LVOT ([92%], *p* < 0.01 and [84%], *p* = 0.01, respectively), AAo ([61%], *p* < 0.001 and [75%], *p* < 0.001), and arch ([53%], *p* < 0.05 and [56%], *p* < 0.001), as shown in Figure 6 and Table 5. Patients who received surgery also demonstrated significantly elevated RF and decreased stasis throughout the entire aorta, most prominently at the arch ([120%], *p* < 0.001 and [-52%], *p* < 0.001).

**TABLE 5:** Parameter differences between BAV patients who did or did not receive aortic surgery following 4D-flow MRI scan.

Parameter	Location									
	LVOT		AAo		Arch		PDAo		DDAo	
	No Surgery (*n* = 49)	Surgery (*n* = 24)	No Surgery (*n* = 49)	Surgery (*n* = 24)	No Surgery (*n* = 49)	Surgery (*n* = 24)	No Surgery (*n* = 49)	Surgery (*n* = 24)	No Surgery (*n* = 49)	Surgery (*n* = 24)
Diameter (mm)	28 ± 4	30 ± 4	38 ± 7	43 ± 6**	25 ± 5	28 ± 4**	22 ± 4	23 ± 3	20 ± 3	21 ± 2
Indexed Diameter (mm/m^2^)	14 ± 2	15 ± 2	19 ± 4	21 ± 4	13 ± 3	14 ± 2	11 ± 2	11 ± 1	10 ± 2	10 ± 1
Peak Velocity (m/s)	1.5 ± 0.5	2.7 ± 1.2**	2.1 ± 0.7	3.7 ± 1.3**	1.3 ± 0.4	2.0 ± 1.0**	1.3 ± 0.7	1.5 ± 1.1	1.1 ± 0.4	1.3 ± 0.6
KE (μJ)	2.0 ± 1.0	3.8 ± 2.3**	1.9 ± 0.8	3.1 ± 1.6**	1.5 ± 0.8	2.3 ± 1.1*	1.6 ± 1.0	1.9 ± 1.3	1.5 ± 0.8	1.8 ± 1.2
FF (mL/cycle)	0.15 ± 0.056	0.17 ± 0.062	0.12 ± 0.031	0.12 ± 0.025	0.14 ± 0.041	0.15 ± 0.034	0.16 ± 0.051	0.16 ± 0.036	0.17 ± 0.049	0.16 ± 0.052
RF (mL/cycle)	0.039 ± 0.031	0.057 ± 0.079	0.033 ± 0.013	0.051 ± 0.016**	0.022 ± 0.013	0.045 ± 0.017**	0.014 ± 0.011	0.033 ± 0.026*	0.009 ± 0.0081	0.028 ± 0.032**
Stasis (%)	26 ± 12	16 ± 8**	25 ± 11	18 ± 9*	38 ± 14	20 ± 10**	41 ± 17	24 ± 14**	43 ± 16	27 ± 13**

LVOT-left ventricular outflow tract, AAo, ascending aorta, Arch - aorta arch; PDAo, proximal descending aorta, DDAo-distal descending aorta, D—Diameter, Di—Dimeter indexed, PV, peak velocity, KE-kinetic energy, FF- forward flow, RF- reverse flow. Values are reported as mean ± stdev. *: *p* < 0.05 and **p < 0.01.

## 4 Discussion

This study uses 4D-flow techniques to evaluate thoracic aorta parametric voxel-by-voxel hemodynamics in the context of BAV disease. Our main findings demonstrate that (Siu and Silversides, 2010) 3D-derived aortic peak velocity, FF, RF, and stasis are significantly altered in BAV patients, and (Sievers and Schmidtke, 2007) Peak velocity, KE, FF, RF, and stasis associate with aortic dilation and referral for surgery. Secondary findings suggest that differences in BAV phenotype and dilation severity impact downstream hemodynamics throughout the thoracic aorta.

### 4.1 Flow Is Abnormal in BAV Patients

Consistent with previous literature (Mathieu et al., 2015; Garcia et al., 2016; Rodríguez-Palomares et al., 2018), we found BAV patients to possess increased peak blood velocity and vessel diameter in the AAo. This is likely due to BAV morphology producing high-velocity jets and BAV patients’ increased propensity for AAo dilatation (Siu and Silversides, 2010). In the current study, Reynolds number indicated presence of turbulence in the ascending aorta. Controls in this study exhibited higher *in vivo* Reynolds numbers than controls in Stalder et al. who reported a range of [3,357–4,528] using prospectively gated 4D flow (Stalder et al., 2011). Some experimental studies reported a range of [2,400–10,000] in the aorta under normal hemodynamic conditions (Keshavarz-Motamed et al., 2014; Ha et al., 2019).

When compared to controls, BAV patients exhibit significantly elevated RF throughout the entire aorta. The RF findings agree well with previous studies that document increased helical flow and vortices within the AAo of BAV patients compared to healthy volunteers (Faggiano et al., 2012; Rodríguez-Palomares et al., 2018; Shan et al., 2019), which is likely a result of the eccentric off-centered jet produced by a BAV (Rodríguez-Palomares et al., 2018). However, RF differences in the distal aorta are less documented. It is possible that minor helical flow persists in the descending aorta, which would maintain elevated RF levels. Regardless of the underlying mechanism, RF levels in the descending aorta have been recently implicated in stroke development. Previous studies have demonstrated the presence of retrograde flow in the descending aorta of cryptogenic stroke patients (Harloff et al., 2010), as well as the theoretical ability of this retrograde flow to carry plaques back towards the brachio-cephalic branches in the arch (Harloff et al., 2007). Harloff et al. estimates that this retrograde flow pattern may account for up to 24% of cryptogenic stroke events (Harloff et al., 2010). Thus, our observed increase in descending aorta RF in BAV patients is an intriguing finding with clinical implications that may warrant future research.

BAV patients were also observed to have significantly reduced blood stasis in all aortic regions (except DDAo) compared to healthy controls. There are very few previous studies which provide context to this finding. Hassanabad et al. (Fatehi Hassanabad et al., 2020) demonstrated that BAV patients, in comparison to healthy volunteers, possessed greater pressure drop throughout the entire thoracic aorta. This may imply an association between pressure drop and stasis. However, in this study we did not evaluate pressure drop and parametric voxel-by-voxel markers were not normalized to arterial pressure.

Lastly, KE levels found in BAV patients were similar to those found in healthy controls, even in the AAo. This is unexpected, as the increased blood jet velocity resulting from a BAV would conceivably lead to greater KE values near the valve. However, previous studies, such as that conducted by Elbaz et al. (Elbaz et al., 2019), have also found similar KE levels between controls and BAV patients in the AAo. To interpret these findings, it is important to note that KE is only one aspect of a fluid’s total energy cost, which also includes turbulent kinetic energy (TKE), viscous energy loss (EL), and heat. It should also be recognized that BAV patients have consistently been shown to exhibit greater aortic TKE and EL, especially in the AAo (Dux-Santoy et al., 2019; Elbaz et al., 2019). Thus, while BAV patients likely possess significantly elevated KE immediately proximal to the valve, it is possible that much of this KE is subsequently lost in the form of EL and TKE due to chaotic helical flow that develops in the AAo. Thus, when we measure the average amount of KE in the entire AAo region over the cardiac cycle–as we do in this study–it is reasonable that KE measurements may be comparable between healthy controls and BAV patients.

### 4.2 Aortic Dilation Is Related to Blood Flow

In the AAo, vessel diameter was negatively associated with regionally-matched stasis and FF. This may be due to the increases in helical flow patterns that accompany AAo dilatation (Hope et al., 2007; Bürk et al., 2012; Dux-Santoy et al., 2019), as helical flow patterns leave less opportunity for forward flow and stasis. While it is important to note that our BAV cohort is older than controls, thus age may be a confounding factor in these associations, our multivariate analysis demonstrated both FF and stasis to be stronger independent predictors of diameter than age.

### 4.3 Ascending Aorta Flow Associates With Surgical Outcomes

No significant difference in aortic diameters was seen between patients who received follow-up surgery and those who did not. Because most of the patients with a surgical event received valvular surgery exclusively, while few patients received AAo replacement surgery, our surgical cohort is mostly characterized by individuals with valvular inefficiencies, rather than aortic aneurysms.

Those who receive surgical intervention exhibited much greater KE, velocity, and RF levels in the proximal aorta. As mentioned above, this is likely a result of the surgical cohort’s level of valvular disease severity. Progression of valvular diseases such as calcification, stenosis, and regurgitation increases blood velocity, vorticity, and retrograde flow within the AAo (Garcia et al., 2019), which would conceivably equate to increased levels of KE, velocity, and RF.

Results also demonstrated significantly lower levels of stasis throughout the entire thoracic aorta in patients who received follow-up surgery compared to patients who did not. Alternatively, the elevated peak blood velocities in the surgical cohort may also explain their lower stasis levels. Either way, stasis appears to be a measure of great difference between patients who received follow-up surgery and patients who did not.

### 4.4 Secondary Findings: Effect of Valve Type and Dilation Severity on Distal Aorta Blood Flow

Previous studies have shown different BAV morphologies and dilation geometries to each possess unique flow characteristics in the AAo (Girdauskas et al., 2012), which may provide great clinical utility in disease severity assessments of BAV patients. Our study has expanded this knowledge by demonstrating that differences in flow patterns between valve phenotypes and dilation severities persist beyond the AAo region into the arch and descending aorta. Thus, it may be of utility for future studies to consider the entire thoracic aorta when characterizing flow patterns of valve types, dilation geometries and aortic arch shape.

### 4.5 Limitations

This study has several limitations. Patients and control cohorts were not age matched; given that age correlated with several parameters of interest, age may be a confounding variable in our findings. Patients were only included if they underwent clinically-ordered MRI examinations for aortic dilation or significant valvular stenosis and/or insufficiency. Accordingly, our patient cohort had a more severe disease phenotype than prior studies. Due to the localized nature of our voxel-by-voxel approach, it cannot be used to assess standard cardiac output. However, cardiac output can be estimated from an analysis plane at the left ventricle outflow tract, in a similar manner to 2D phase-contrast. As a cross-sectional design looking at the associations of parameters at a single point in time, no causative relationships can be determined from our analyses; future longitudinal studies are needed. Valve type, dilation severity, and surgical outcome cohort sizes were modest, and thus findings drawn from their analyses are limited. The parametric hemodynamic parameters presented in this study were measured over the entire cardiac cycle, rather than at systole or diastole. This reduces the sensitivity of our results and may overlook important phenomena occurring at specific time points in the cardiac cycle. We did not monitor progression rates over time (i.e. between several serial visits) using parametric voxel-by-voxel markers. Similarly to wall shear stress (van Ooij et al., 2016), voxel-by-voxel markers can be used for generating hemodynamic atlases, which allow a personalized evaluation of disease progression, as recently reported by Soulat’s et al. (Soulat et al., 2021).

Lastly, WSS was not included in the analysis for the presented cohort. It may be relevant to explore the association between 3D parametric voxel-by-voxel markers, axial/circumferential WSS (Rodríguez-Palomares et al., 2018), and in-plane rotational flow (Dux-Santoy et al., 2019). Most WSS studies usually report WSS magnitude (Mahadevia et al., 2013; Guzzardi et al., 2015; van Ooij et al., 2016; van Ooij et al., 2017) rather than the WSS vector decomposition. Recent studies reported that increased in-plane rotational flow and higher axial/circumferential WSS may explain aortic dilation morphotypes (Rodríguez-Palomares et al., 2018; Dux-Santoy et al., 2019).

## 5 Conclusion

This study used novel measurement techniques to comprehensively explore thoracic aortic hemodynamics in the context of BAV disease. BAV patients present significantly altered 3D-derived hemodynamics throughout the thoracic aorta compared to healthy controls, some of which are associated with measures of aortic dilation and the need for surgery. Further longitudinal studies are needed to explore these flow parameters in relation to BAV aortopathy, especially stasis and reverse flow, in the effort to provide improved clinical management of BAV patients.

## Data Availability

The raw data supporting the conclusion of this article will be made available by the authors, without undue reservation.
